# Multi-Dimensional Uniform Initialization Gaussian Mixture Model for Spar Crack Quantification under Uncertainty

**DOI:** 10.3390/s21041283

**Published:** 2021-02-11

**Authors:** Qiuhui Xu, Shenfang Yuan, Tianxiang Huang

**Affiliations:** Research Center of Structural Health Monitoring and Prognosis, State Key Lab of Mechanics and Control of Mechanical Structures, Nanjing University of Aeronautics and Astronautics, Nanjing 210016, China; qhx@nuaa.edu.cn (Q.X.); tianxiang.huang@nuaa.edu.cn (T.H.)

**Keywords:** structural health monitoring, guided wave, Gaussian mixture model, crack quantification, uncertainty, time-varying conditions

## Abstract

Guided Wave (GW)-based crack monitoring method as a promising method has been widely studied, as this method is sensitive to small cracks and can cover a wide monitoring range. Online crack quantification is difficult as the initiation and growth of crack are affected by various uncertainties. In addition, crack-sensitive GW features are influenced by time-varying conditions which further increase the difficulty in crack quantification. Considering these uncertainties, the Gaussian mixture model (GMM) is studied to model the probability distribution of GW features. To further improve the accuracy and stability of crack quantification under uncertainties, this paper proposes a multi-dimensional uniform initialization GMM. First, the multi-channel GW features are integrated to increase the accuracy of crack quantification, as GW features from different channels have different sensitivity to cracks. Then, the uniform initialization method is adopted to provide more stable initial parameters in the expectation-maximization algorithm. In addition, the relationship between the probability migration index of GMMs and crack length is calibrated with fatigue tests on prior specimens. Finally, the proposed method is applied for online crack quantification on the notched specimen of an aircraft spar with complex fan-shaped cracks under uncertainty.

## 1. Introduction

Structural health monitoring (SHM) technology has gradually developed from basic theoretical research in the laboratory to real aircraft applications recently [1,2,3,4]. Among them, the guided wave (GW) based SHM method has been widely studied because this method can cover a wide monitoring range and it is sensitive to small damage [5,6,7,8,9,10,11]. In addition, the GW-based SHM method can also be applied on-line both for damage monitoring and impact event monitoring.

Fatigue crack is one of the primary damage types in engineering structures. Accurate fatigue crack quantification is of great significance for ensuring the safety and reliability of aircraft structures. Moreover, it can realize condition-based maintenance and extend structure service life. However, there are some obstacles to crack monitoring in real engineering applications. First, initiation and growth of crack are affected by various uncertainties [12,13], such as inhomogeneity of material, scatter of mechanical properties of the material, the randomness of cracking process, and quality of manufacturing, etc. These uncertain factors lead to the scatter of both crack size and fatigue lifetime. Second, various time-varying conditions introduce uncertainty effects on GW features. These time-varying conditions include random dynamic load, varying environmental temperature, changing structural boundary conditions, noise, etc. GW features sensitive to the crack are also usually sensitive to time-varying conditions. Therefore, the uncertain and random variations of the GW features caused by the time-varying conditions lead to lower reliability and stability for crack monitoring in real engineering applications.

Considering these uncertainties, some probability statistical models are widely studied for fatigue crack diagnosis and prognosis recently. Probability statistical models can be introduced to construct a mathematical model of damage features to estimate the damage of structures. Yuan and Mei et al. [14] proposed the GW-Hidden Markov model based method to achieve a probabilistic evaluation of the propagation state of cracks. Uncertainties such as changing load and structural boundary conditions were considered in this research. For crack prognosis, Chen et al. [15] improved the particle filter based methods. In addition, some other probability statistical models, such as the stochastic global model [16] and Gaussian process [17] are also able to characterize uncertainties of GW signals. These probabilistic statistical models focus on addressing different aspects of crack diagnosis and prediction under uncertainty. However, more research is still needed for real engineering applications. 

In recent years, the Gaussian mixture model (GMM) has been proven to be an effective approach to model the complicated probability distributions of GW features. GMM is a data-driven model without physical modeling. It can model arbitrary probability distributions by decomposing a non-Gaussian distribution into a combination of a finite number of Gaussian components (GCs) based on unsupervised learning [18]. Yuan and Qiu et al. [19] proposed the GW-GMM based method to model the probability characteristic of GW features under time-varying conditions. This method is validated in the full-scale aircraft fatigue test. Chakraborty et al. [20] proposed a Dirichlet process GMM and it was validated on an aluminum compact tension specimen for crack monitoring subjected to variable-amplitude cyclic load. Banerjee et al. [21] applied the GMM for crack propagation monitoring under the condition of sudden temperature changes. All these researches have proved the potential to apply the GMM to deal with the time-varying problem. However, deep research still needs to be performed concerning further improve the accuracy and stability of crack quantification under uncertainties.

Some structures of aircraft are very complex, and their cracking forms are also complex. For example, the crack form is complex fan-shaped at the notch-edge in an aircraft landing gear spar. There are cracks in multiple directions at the same crack initiation point. At this time, a single channel cannot meet the accuracy requirements of crack quantification. Some researchers reported that different piezoelectric (PZT) excitation-sensing channels can have different sensitivity to damage [22]. Therefore, it is necessary to fuse multi-channel GW features for more accurate crack quantification.

Furthermore, the GMM construction results are influenced by the initial parameters. The parameters of the GMM can be obtained by using the expectation-maximization (EM) algorithm, which is sensitive to the initial value [23]. If the initialization parameters are not set properly, it is easy to make the EM algorithm converge to a relatively poor local optimal solution. EM algorithm initialized by the *k*-means method is widely used to construct a GMM [18]. However, the centroids are chosen randomly in the *k*-means method, even with the same training data, *k*-means can lead to different results and thus provide different GMM initial parameters.

Thus, this paper proposes a multi-dimensional uniform initialization Gaussian mixture model (MdUI-GMM) to improve the accuracy and stability of on-line crack quantification under uncertainty. The multi-dimensional GMM is established by integrating GW features of multiple channels. In this paper, the damage index (DI) based on normalized correlation moment (NCM) is selected as the GW feature. Considering the different sensitivity of different PZT excitation-sensing channels, it is possible to increase the accuracy of crack quantification by integrating the GW features of each channel. Moreover, a uniform initialization method is adopted to improve the stability of multi-dimensional GMM. Compared with the traditional *k*-means initialization method, the stability of modeling results is improved. Finally, the proposed method is applied for online crack quantification on notched specimens of an aircraft spar with complex fan-shaped cracks under uncertainty. 

This paper is organized as follows. Section 2 proposes the MdUI-GMM based crack quantification method, including multi-channel fusion GW features extraction, the modeling process of MdUI-GMM, and the migration measuring method. The implementation process of the MdUI-GMM based crack quantification process is summarized as well. In Section 3, the notched specimen of an aircraft spar fatigue test is introduced, and the multi-channel GW features acquired under dynamic load are given and discussed. Then, crack quantitative calibration results by the MdUI-GMM and crack monitoring results are shown. In addition, comparisons between single-channel and multi-channel fusion measurement are discussed. Finally, conclusions are given in Section 4.

## 2. MdUI-GMM Based Crack Quantification Method

In this section, the multi-channel fusion GW features extraction is described first. Then, the modeling process and the migration measuring method of MdUI-GMM are proposed. Finally, the implementation process of the MdUI-GMM based crack quantification process is summarized as well.

### 2.1. Multi-Channel Fusion GW Features Extraction

There are great uncertainties in the crack initiation point and crack propagation direction of each specimen because of the scatter of the manufacturing process and external fatigue load, etc. It is not reliable for crack quantification only based on a single excitation-sensing channel. Because different PZT excitation-sensing channels have different sensitivity to crack, it is possible to increase the accuracy of crack quantification by integrating multi-channel GW features.

The GW-based SHM is deemed as one of the most appealing methods due to its merits including sensitivity to small damage and the ability to monitor a region when forming a sensor network. Damage such as cracks within a structure can cause scattering and energy absorption of GW propagating through the structure. So the crack can be detected when it is located in the monitoring area which is defined by the sensor network. Generally speaking, when performing the GW based SHM technology, a lot of GW signals can be acquired during the online damage monitoring process and the corresponding GW features can be extracted using different kinds of signal processing methods. The GW features can be extracted in the time domain, frequency domain, and time-frequency domain [24,25,26]. The DI based on NCM [27] is used in this article. *DI*_NCM_ can be calculated by Equation (1), which can be used to obtain the variation of GW signals phase and amplitude.

(1) $$DI_{NCM}=\frac{\int_{\tau =t_{0}}^{\tau =t_{1}}\tau^{2}\left|r_{bb}\left(\tau \right)\right|d\tau -\int_{\tau =t_{0}}^{\tau =t_{1}}\tau^{2}\left|r_{bm}\left(\tau \right)\right|d\tau }{\int_{\tau =t_{0}}^{\tau =t_{1}}\tau^{2}\left|r_{bb}\left(\tau \right)\right|d\tau },$$

where

(2) $$r_{bb}\left(\tau \right)=\int_{t_{0}}^{t_{1}}b\left(t\right)b\left(t-\tau \right)dt,r_{bm}\left(\tau \right)=\int_{t_{0}}^{t_{1}}b\left(t\right)m\left(t-\tau \right)dt,$$

where ***b***(*t*) and ***m***(*t*) represent baseline signal and monitoring signal respectively, *t*_0_ and *t*_1_ are the start and stop time corresponding to the selected signal segment. In this paper, the baseline signal ***b***(*t*) is the average of the first 10 dynamic load signals at the initial stage of fatigue loading when the structure is in a healthy state.

### 2.2. Modeling Process of MdUI-GMM

The GW signals acquired under aircraft structure service conditions can be considered as a mixture of uncertain changes. Consequently, a GW feature can be considered as a random variable. Let **X** = [***X***_1_, ***X***_2_, …, ***X****_r_*, …, ***X****_R_*] be a GW feature sample set composed of *R* independent features that are obtained from *R* GW signals. ***X****r* denotes a *d*-dimensional GW feature in the sample set, where ***X****r* = [*DI*_1_, *DI*_2_, …, *DI_d_*] ^T^ and *r* = 1, 2, …, *R*.

Generally, the variation of GW features introduced by time-varying conditions is random and complex. Consequently, when using a single probability density function to describe the distribution of the GW feature sample set, it may yield unexpected and inaccurate results. Therefore, the mixture probability model is applied to address this problem. The joint probability density function (PDF) of the GW feature sample set can be approximately modeled by a GMM which is considered as a finite weighted sum of GCs. Similar to [18,28], the GMM is expressed as Equation (3):

(3) $$\Phi \left(X|\mu ,\Sigma \right)=\sum_{i=1}^{C}w_{i}\Phi_{i}\left(X_{r}|\mu_{i},\Sigma_{i}\right),$$

where *C* is the number of GCs, *i* = 1, 2, …, *C*. **μ***_i_* and **Σ***_i_* are the mean and the covariance matrix of the *i*th GC. *w_i_* is the mixture weight of the *i*th GC. The PDF of each GC Φ*_i_* is a *d*-dimensional Gaussian function which is expressed as Equation (4) [15]

(4) $$\Phi_{i}\left(X_{r}|\mu_{i},\Sigma_{i}\right)=\frac{1}{{\left(2\pi \right)}^{\frac{d}{2}}\sqrt{\left|\Sigma_{i}\right|}}e^{-\frac{1}{2}{\left(X_{r}-\mu_{i}\right)}^{T}\Sigma_{i}^{-1}\left(X_{r}-\mu_{i}\right)},$$

In this paper, the GW features of three channels are extracted to construct multi-dimensional GMM, where *d* = 3.

The value of *w_i_*, **μ***_i_*, and **Σ***_i_* can be obtained based on the sample set by using the EM algorithm. To ensure the stability of training, a uniform initialization process instead of the *k*-means initialization method is performed. The main modeling process of MdUI-GMM is as follows:

Step 1: Before the EM algorithm begins, the uniform weights and posterior probabilities are given for each GC, as indicated in Equations (5) and (6)

(5) $$P_{ri,0}=\frac{1}{C},$$

(6) $$w_{i,0}=\frac{1}{C},$$

where *C* is the number of GCs, *i* = 1, 2, …, *C*. *P_ri_*_,0_ represents the probability that the initialized *r*th GW feature belongs to the *i*th GC and $\sum_{i=1}^{C}P_{ri,0}=1$. *w_i_*_,0_ is the initialized mixture weight of the *i*th GC and $\sum_{i=1}^{C}w_{i,0}=1$. Because the total number of GC is *C*. Therefore, the posterior probabilities *P*_ri_ and mixture weight *w*_i_ can be initialized uniformly, as shown in Equations (5) and (6). Based on the uniform initial values of the *P_ri_*_,0_ and *w_i_*_,0_, the parameters of GMM can be calculated iteratively by the EM algorithm in step 2 and step 3. Since this initialization is only related to the number of GC, a stable initialization result can be obtained after the number of GC is determined.

Step 2: Based on the uniform initial values of the weights and posterior probabilities, the mean vectors and covariance matrices of UIGMM are calculated using Equations (7) and (8)

(7) $$\mu_{i}{}_{,t}={\left[Rw_{i,t-1}^{}\right]}^{-1}\sum_{r=1}^{R}P_{ri,t-1}^{}\cdot X_{r},$$

(8) $$\Sigma_{i,t}={\left[Rw_{i,t-1}\right]}^{-1}\sum_{r=1}^{R}P_{ri,t-1}^{}\left[X_{r}-\mu_{i,t}\right]{\left[X_{r}-\mu_{i}{}_{,t}\right]}^{T},$$

where *t* is the number of iterations and *R* is the total number of GW features used to construct GMM. *w_i_*_,t−1,_ and *P_ri_*_,t−1_ represent the weight and posterior probability of the last iteration, respectively.

Step 3: The posterior probability and weight of GCs are updated according to Equations (9) and (10)

(9) $$P_{ri,t}=\frac{w_{i,t}{}_{-1}\Phi (X_{r},\mu_{i,t},\Sigma_{i,t})}{\sum_{j=1}^{C}w_{j,t-1}\Phi (X_{r},\mu_{j,t},\Sigma_{j,t})},$$

(10) $$w_{i,t}=\frac{1}{R}\sum_{r=1}^{R}P_{ri,t},$$

where *P_ri_*_,t_ is the probability that the *r*th GW feature belongs to the *i*th GC in the *t*th iteration.

Step 4: Repeat Step 2 and Step 3 until the log-likelihood value converges.

(11) $$L=\log\Phi \left(X|w,\mu ,\Sigma \right)=\log\prod_{i=1}^{R}\Phi \left(X_{r}|w,\mu ,\Sigma \right)=\sum_{r=1}^{R}\log\left[\sum_{i=1}^{C}w_{i}\Phi_{i}(X_{r}|\mu_{i},\Sigma_{i})\right],$$

(12) $$Log\left(L_{t}\right)-Log\left(L_{{}^{t-1}}\right)<\varepsilon ,$$

*L_t−_*_1_ is the value of the log-likelihood function in the previous iteration step, *L_t_* is the log-likelihood function value of the current step. The value of *ε* is set to be 1 × 10^−10^.

### 2.3. On-Line Migration Measuring Method of the MdUI-GMM

The migration measuring result between the on-line monitoring MdUI-GMM and the baseline MdUI-GMM is defined as the probability migration index (PMI). If the monitored structure keeps healthy, the result of PMI will be maintained at a low level. If a crack occurs in the structure and propagates continuously, a cumulative migration trend will appear in both the MdUI-GMM and the PMI. Based on the result of PMI, reliable damage monitoring can be realized. Considering that the MdUI-GMM is composed of many GCs, a Kullback-Leibler (KL) divergence [29] based method is used to calculate the PMI. 

The PDF of the baseline GMM and the *n*th monitoring GMM are Φ^0^ and Φ*^n^*, respectively. The KL divergence-based PMI can be calculated by Equation (13).

(13) $$D_{KL}\left(\Phi^{0}\|\Phi^{n}\right)=\frac{1}{2}[tr\left({\left(\Sigma^{n}\right)}^{-1}\Sigma^{0}\right)+{\left(\mu^{n}-\mu^{0}\right)}^{T}{\left(\Sigma^{n}\right)}^{-1}\left(\mu^{n}-\mu^{0}\right)-d-\ln\left(\frac{\left|\Sigma^{n}\right|}{\left|\Sigma^{0}\right|}\right)],$$

where *d* is the dimension of the GW feature vector. **μ^0^** and **Σ^0^** represent the weighted sum of the mean and covariance matrices in the baseline GMM, respectively. **μ*^n^*** and **Σ*^n^*** represent the weighted sum of the mean and covariance matrices in the *n*th monitoring GMM, respectively. tr is the matrix trace.

### 2.4. The MdUI-GMM Based Crack Quantification Process

The effect of time-varying conditions on GW features is random and uncertain. However, there is a cumulative migration trend in the effect of cracks on GW features. Considering this, the GMM is used to model the probability distribution of GW features and suppress the influence of uncertainty. If the structure is in a healthy state or if the cracks are not extended, each GC of the GMM can cover the random distribution of the GW features due to environmental changes. Time-varying conditions (e.g., random changes with dynamic load, or temperature, etc.) do not change the form of the GMM distribution. If the crack propagates, the form of the distribution of the GCs in the GMM undergoes a cumulative migration change. That is, under the influence of cracks, The PDF of monitoring GMM undergoes a cumulative migration change compared to the PDF of the baseline GMM.

**Figure 1:**
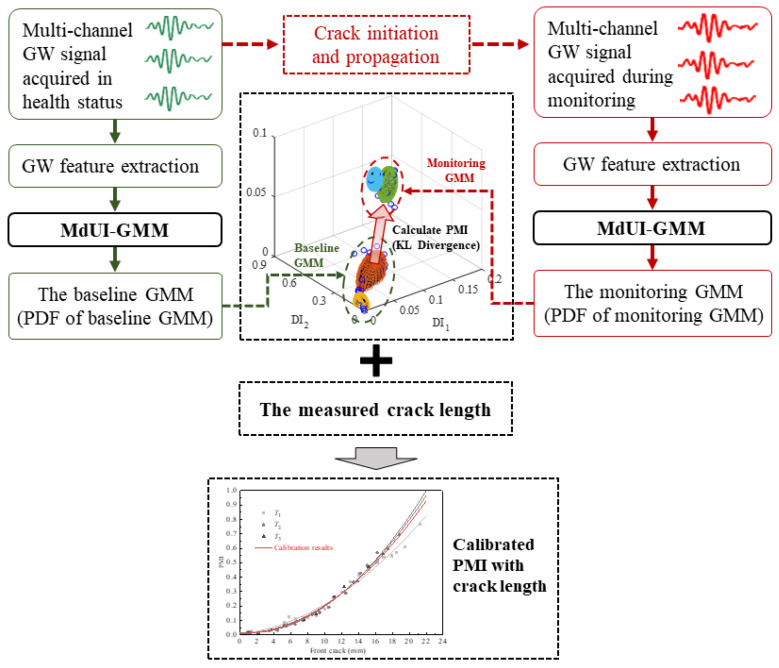
The process of calibrating probability migration index (PMI) with crack length using prior specimens.

The calibrating process of the MdUI-GMM based crack quantification method is shown schematically in Figure 1. The process of calibrating PMI with crack using prior specimens includes three parts:

Part 1 is the baseline MdUI-GMM. GW signal acquisition is performed under time-varying conditions when the structure is in a healthy state. GW features calculated by Equation (1) are extracted to construct a GW baseline sample set. After that, the parameters of MdUI-GMM, including weight, mean, and covariance matrix, are trained by an EM algorithm with a uniform initialization process. Finally, the PDF of the baseline GMM can be obtained.

Part 2 is online monitoring MdUI-GMM. Once a GW signal is acquired during the online damage monitoring process, new GW features can be obtained, and the GW feature sample set is updated first. The updating rule is called first in and first out which means that the new feature is added to be the last (newest) feature and the first (oldest) feature is removed. Hence, the number of GW features in the GW feature sample set is consistent. Then, the parameters of monitoring GMM are trained by an EM algorithm with a uniform initialization process. Finally, the PDF of monitoring GMM can be obtained.

Part 3 is crack quantification calibration. When a certain number of load cycles is reached, the fatigue test is paused to measure the crack length using an electron microscope and scale lines on one surface of the specimen. After crack length measurement is completed, fatigue testing is resumed. By calculating KL divergence between the baseline GMM in part 1 and the monitoring GMM in part 2, PMI can be obtained. Finally, in combination with the measured crack length, a calibration between PMI and crack length can be performed.

**Figure 2:**
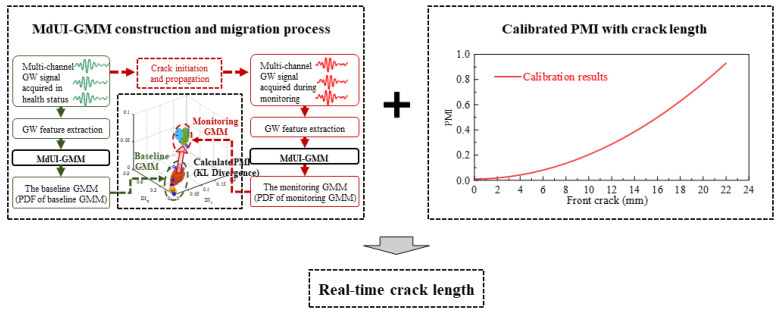
Online crack quantification of the new specimen.

The process of online crack quantification of the new specimen is shown schematically in Figure 2. Under time-varying conditions, the GW features of the new specimen are obtained online to construct the online monitoring MdUI-GMM and then PMI can be calculated. Finally, according to the quantitative calibration relationship between PMI and crack length in Part 3, the real-time crack length can be calculated directly.

## 3. Validation on the Notched Specimen of an Aircraft Spar under Fatigue Load

In this section, the fatigue test of notched specimens of an aircraft spar is introduced. Then, the multi-channel GW features acquired under dynamic load are given and discussed. After that, crack quantitative calibration results of prior specimens by the MdUI-GMM, and monitoring results are shown.

### 3.1. Experimental Setup

**Figure 3:**
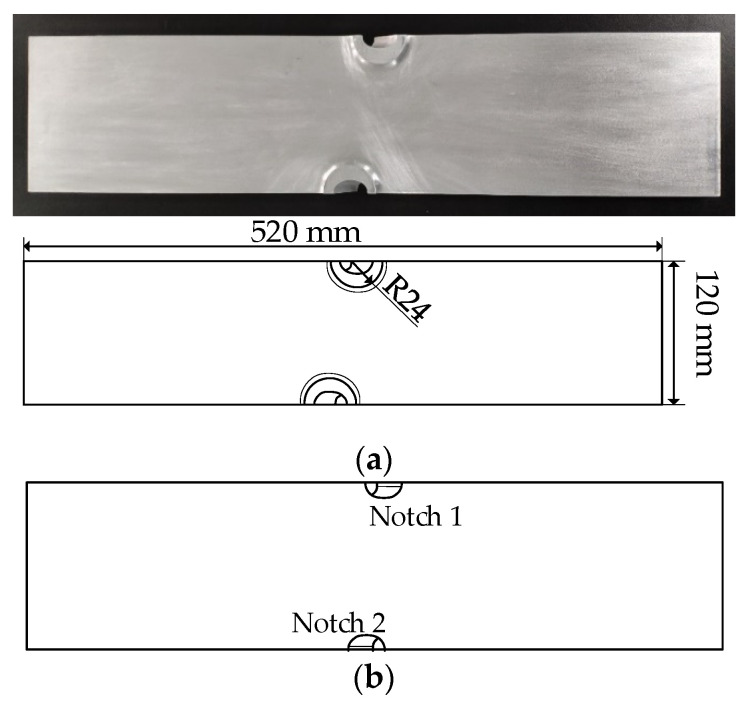

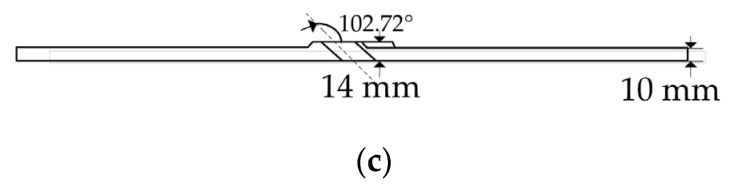
The geometry of the specimen. (**a**) Front view. (**b**) Back view. (**c**) Side view.

A typical load-transmitting component in aircraft structures is studied in this paper as illustrated in Figure 3. The thickness of the specimen is 10 mm. There are two oblique notches with a radius of 8 mm on both sides of the length direction. The oblique axis of the notch on both sides is 102.72°. To increase the strength of the specimen, two bosses are in the thickness direction. The radius of the outer edge of the boss is around 20 mm, and the height is around 4 mm. Four specimens, with the same size and material parameters, are tested and marked as *T*_1_–*T*_4_, respectively. The first three specimen *T*_1_–*T*_3_ are used as prior specimens for quantitative calibration, and *T*_4_ is used for testing.

The material of the specimen is aluminum alloy 7A04 T7351. Table 1 lists the mechanical properties of aluminum alloy 7A04.

**Figure 4:**
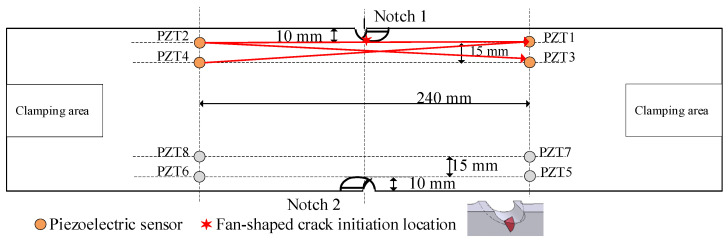
Piezoelectric (PZT) layers layout.

PZT layers are attached to each specimen and used as the actuators and the sensors, whose positions are illustrated in Figure 4. PZT2 and PZT4 are used as the actuators. PZT1 and PZT3 are used as sensors. The distance between Channel 2-1 and Channel 4-3 is 15mm. Because there are two notches in the specimen, cracks may be initiated at both notches, so another four PZT layers are also arranged at the other notch. When the crack starts from Notch 1, the GW features extracted from channel 2-1, channel 2-3, and channel 4-1 will be integrated to construct multi-dimensional GMM. When the crack starts from Notch 2, the GW features extracted from channel 6-5, channel 6-7, and channel 8-5 will be integrated to construct multi-dimensional GMM.

**Figure 5:**
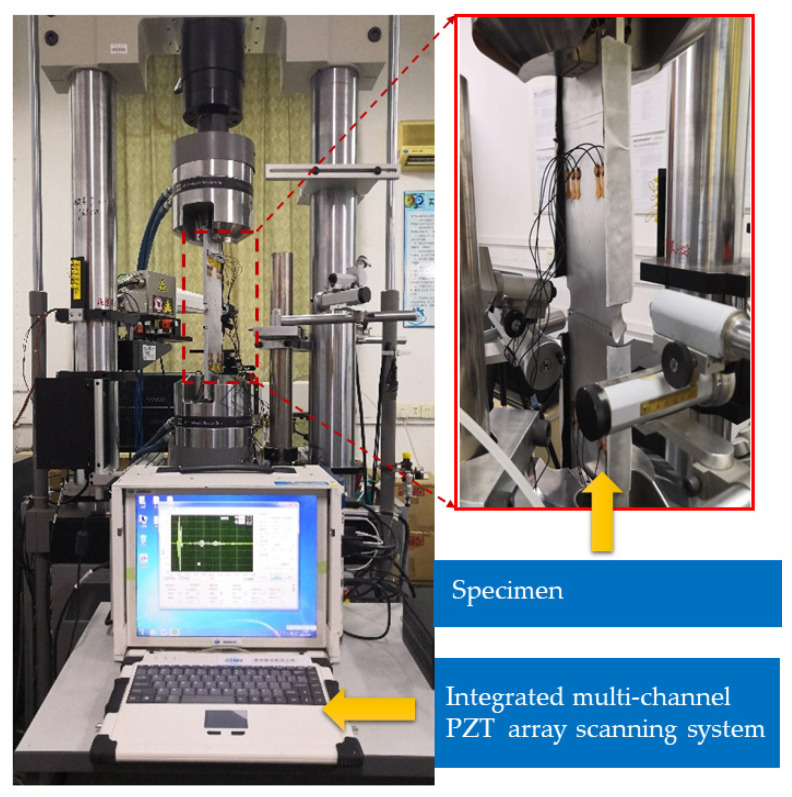
The experimental setup.

The experimental setup is illustrated in Figure 5. An MTS electro-hydraulic servo tensile machine is used to apply the variable amplitude fatigue load. The fatigue load spectrum is generated by using the in-service flight data to simulate different flight modes of the aircraft such as takeoff, level flight, overloading, landing, on-ground, etc. A digital microscope is applied to measure crack length. The multi-channel PZT array scanning system developed by the authors’ group is employed to perform the active GW-based structural health monitoring. A three-cycle tone-burst signal with the center frequency of 110 kHz and ±70 V amplitude is used as the excitation signal. The sampling frequency is set to be 50 M samples s^−1^, where the high time resolution of the GW signals enables accurate damage feature extraction. 

Based on the in-service experience, the edge fan-shaped cracks of oblique notches are the main damage forms, because the specimen is thick, and it is the primary load-bearing structure of the aircraft. There are front crack and side crack at the same crack initiation point. The front crack propagates along the width direction in the front of the specimen. The side crack extends along the thickness direction. The crack starts from a certain point in the notch and gradually propagates to the inside of the specimen.

The front crack and the side crack have the same crack initiation point. The angle between the front crack and the side crack is approximately 90°. Taking the crack initiation point as the center of the circle, the length of the front crack and the side crack is regarded as the semi-major axis and the semi-minor axis of the ellipse. The front crack length and the side crack length are recorded as *a* and *b*, respectively. The damaged area A can be calculated by Equation (14).

(14) $$A=\frac{1}{4}\pi ab,$$

Generally, with the propagation of the side cracks, the thickness direction is gradually penetrated through, and then the fracture rapidly expands along the width direction, resulting in the fracture of the specimen. During the loading process of the fatigue test, cracks of the *T*_1_–*T*_4_ are all initiated at Notch 1. Due to the difference in material properties and external load, the crack initiation position of each specimen is different.

**Figure 6:**
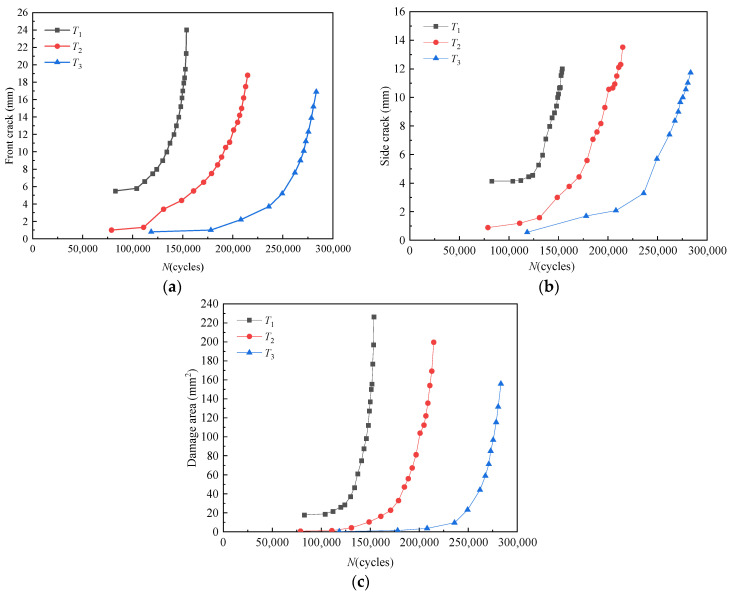
Relationship between damage degree and load cycles. (**a**) Front crack length. (**b**) Side crack length. (**c**) Damage area.

To quantitatively monitor the crack growth, the crack length is measured by a digital microscope. Figure 6 shows the relationship between the front crack length, the side crack length, and the damaged area with the number of load cycles.

It can be seen that the growth rate of the front crack and side crack is relatively slow at first. With the increase of load cycle number and crack length, the crack growth rate increases. However, the uncertainty leads to the scatter of both crack size and fatigue lifetime.

### 3.2. GW Features Extraction under Uncertainty

**Figure 7:**
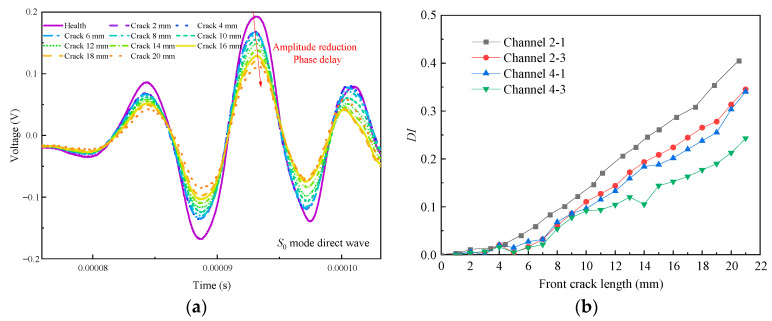
Guided wave (GW) signal and multi-channel GW features without time-varying conditions. (**a**) Typical *S*_0_ mode direct wave signal obtained from *T*_2_. (**b**) Multi-channel GW features of *T*_2_.

Figure 7a shows the typical *S*_0_ mode direct wave signal obtained from *T*_2_ channel 2-1 without time-varying conditions. When the excitation center frequency is 110 kHz, the complete *S*_0_ mode direct wave can be obtained. When there is no crack in the specimen, the peak amplitude of the *S*_0_ mode direct wave is 193.7 mV and the corresponding phase is 92.5 μs. When the front crack extends to 20 mm, the peak amplitude of the *S*_0_ mode direct wave is 109.4 mV and the corresponding phase is 93.1 μs. For every 2 mm crack propagation, the peak amplitude of the *S*_0_ mode direct wave decreases by about 20 mV. Therefore, without the influence of time-varying conditions, the crack can be monitored by the variation of GW signals phase and amplitude. As shown in Figure 7b, without environmental effects, there are differences in the GW features acquired by different channels when only the effects of cracks are considered. Considering that different channels have different sensitivity to cracks, it is possible to increase the accuracy of crack quantification by integrating multi-channel GW features. The GW feature extracted in this paper is *DI*_NCM_ by Equation (1).

However, the structure is rarely only affected by static load without time-varying conditions in real engineering applications. Therefore, crack monitoring under time-varying dynamic load will be discussed. The direct wave signal segment of *S*_0_ mode is intercepted to calculate the DI under dynamic load by the Equation (1). *t*_0_ and *t*_1_ of the signal segment are 0.8 × 10^−4^ s and 1.05 × 10^−4^ s, respectively. The baseline signal ***b***(*t*) is the average of the first 10 dynamic load signals at the initial stage of fatigue loading when the structure is in a healthy state.

**Figure 8:**
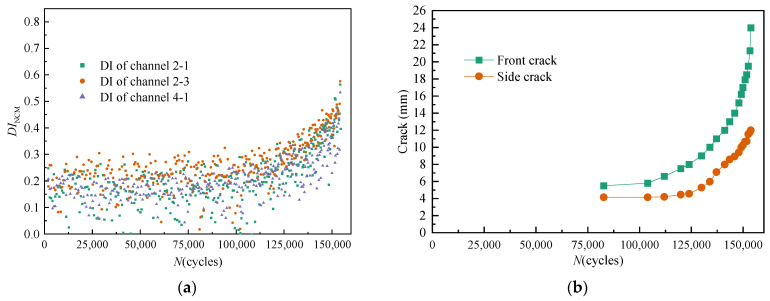
Variation of multi-channel damage indexes (DIs) and crack length of *T*_1_ under dynamic fatigue load. (**a**) Multi-channel *DI*_NCM_. (**b**) Crack length.

For *T*_1_, all DIs extracted from channel 2-1, channel 2-3, and channel 4-1 under time-varying dynamic load are illustrated in Figure 8a. The variation of the front crack length and the side crack length with the number of load cycles is illustrated in Figure 8b.

**Figure 9:**
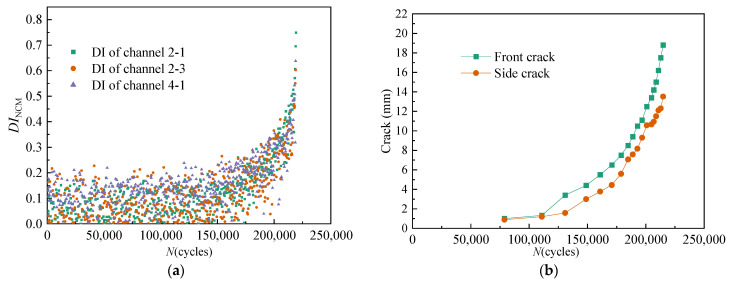
Variation of multi-channel DIs and crack length of *T*_2_ under dynamic fatigue load. (**a**) Multi-channel *DI*_NCM_. (**b**) Crack length.

For *T*_2_, all DIs extracted from channel 2-1, channel 2-3, and channel 4-1 under time-varying dynamic load are illustrated in Figure 9a. The variation of the front crack length and the side crack length with the number of load cycles is illustrated in Figure 9b.

**Figure 10:**
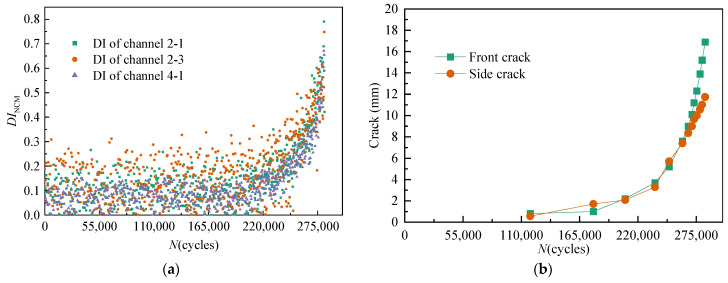
Variation of multi-channel DIs and crack length of *T*_3_ under dynamic fatigue load. (**a**) Multi-channel *DI*_NCM_. (**b**) Crack length.

For *T*_3_, all DIs extracted from channel 2-1, channel 2-3, and channel 4-1 under time-varying dynamic load are illustrated in Figure 10a. The variation of the front crack length and the side crack length with the number of load cycles is illustrated in Figure 10b.

As shown in Figure 8, Figure 9 and Figure 10, the effect of time-varying conditions on GW features is random and uncertain. Therefore, the traditional DI method has limitations in crack quantification. However, there is a cumulative migration trend in the effect of cracks on GW features. 

### 3.3. Establishment and Stability of MdUI-GMM

Considering that the traditional DI method has limitations in crack quantification under time-varying conditions. In this section, MdUI-GMM is used to model the probability distribution of GW features and suppress the influence of uncertainty.

#### 3.3.1. The Construction Process of MdUI-GMM

**Figure 11:**
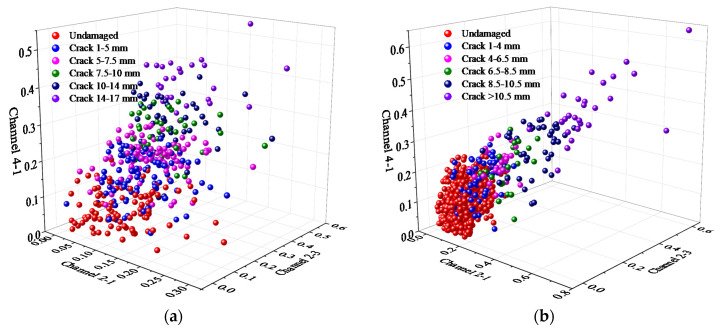
Multi-dimensional GW feature sample set. (**a**) *T*_1_. (**b**) *T*_2_.

For *T*_1_ and *T*_2_, all the DIs extracted from channel 2-1, channel 2-3, and channel 4-1 are used as the coordinate values of the three dimensions to draw the spatial scatter diagram, which is shown in Figure 11. It can be seen that DIs of different crack lengths are overlapped seriously and the spatial distribution of the GW feature sample set has a cumulative migration trend under time-varying dynamic load.

**Figure 12:**
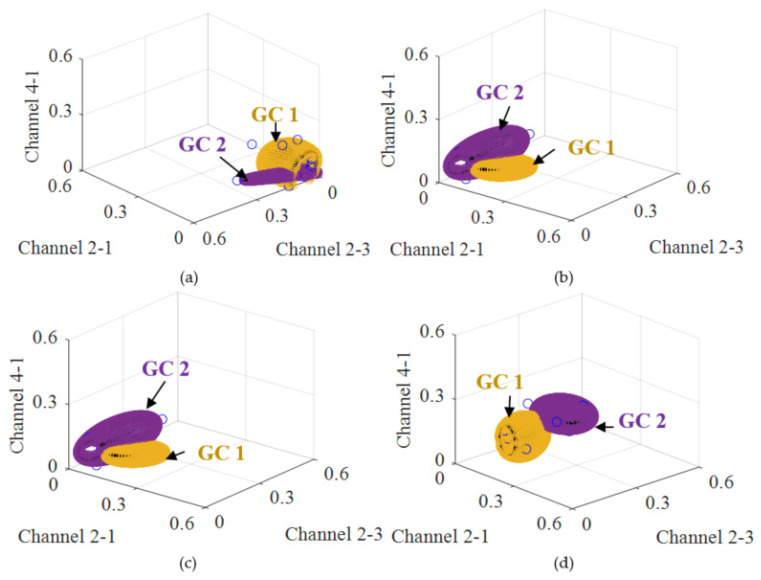
Multi-dimensional uniform initialization Gaussian mixture model (MdUI- GMM) migration process of the typical specimen. (**a**) Baseline GMM. (**b**) Monitoring GMM: crack length = 3 mm. (**c**) Monitoring GMM: crack length = 6 mm. (**d**) Monitoring GMM: crack length = 9 mm. (The blue points indicate the GW features acquired from the multi-channel. The yellow regions indicate the PDF of GC 1. The purple regions indicate the PDF of GC 2.).

The first 30 multi-dimensional GW features extracted from 30 dynamic GW signals are used as a GW baseline sample set to construct a baseline MdUI-GMM. Once a GW signal is acquired during the online damage monitoring process, new GW features can be obtained, and the GW feature sample set is updated first. Then, the online monitoring MdUI-GMM can be constructed by the EM algorithm with a uniform initialization process. Finally, PMI can be calculated. The number of GCs is set to be *c* = 2 to cover the GW feature sample set. The total number of GW feature sample set used to construct MdUI-GMM is *R* = 30. The baseline MdUI-GMM of the typical specimen is shown in Figure 12a. Based on the migration method, some typical on-line monitoring MdUI-GMMs accompanying the crack propagation are shown in Figure 12b, c, d, respectively. The yellow regions indicate the PDF of GC 1 and the purple regions indicate the PDF of GC 2. These yellow and purple ellipsoids represent contour plots of 3-dimensional Gaussian distributions. The parameters of the ellipsoids are determined by the mean and covariance of the Gaussian distribution which account for 95% of the sample set (two standard deviations from the mean). The blue points indicate the GW features acquired from the multi-channel under dynamic loading. The *x*-axis coordinates of the blue points are *DI*_NCM_ of channel 2-1. The *y*-axis coordinates are *DI*_NCM_ of channel 2-3. The *z*-axis coordinates are *DI*_NCM_ of channel 4-1. *DI*_NCM_ can be calculated by Equation (1).

When the crack propagates, the form of the distribution of the GCs in the GMM undergoes a cumulative migration change. That is, the PDF of monitoring GMM undergoes a cumulative migration change compared to the PDF of the baseline GMM. The PMI between monitoring GMM and baseline GMM can be calculated by Equation (13). In combination with the measured crack length, a calibration can be performed, which will be discussed in Section 3.4.

#### 3.3.2. Stability Comparison of Different Initialization Methods

To analyze the stability of the proposed MdUI-GMM, a standard deviation (SD)-based statistical analysis method is applied. First, MdUI-GMM is performed 100 times with the training dataset of the *T*_1_. In the case of multi-dimensional (3D) GMM, each GC corresponds to a weight, and the mean value of each GC is a vector of 3 × 1 and the covariance is a matrix of 3 × 3. The *SD*s of the MdUI-GMM parameter sets are then calculated according to Equations (15)–(17).

(15) $$SD_{w}=\sqrt{\frac{1}{100}\sum_{i=1}^{100}{\left(w_{c}^{i}-{\bar{w}}_{c}\right)}^{2}},$$

(16) $$SD_{\mu }=\frac{1}{3}\left(\sum_{j=1}^{3}\sqrt{\frac{1}{100}\sum_{i=1}^{100}{\left(\mu_{c,j}^{i}-{\bar{\mu }}_{c,j}\right)}^{2}}\right),$$

(17) $$SD_{\Sigma }=\frac{1}{9}\left(\sum_{k=1}^{3}\left(\sum_{j=1}^{3}\sqrt{\frac{1}{100}\sum_{i=1}^{100}{\left(\Sigma_{c,kj}^{i}-{\bar{\Sigma }}_{c,kj}\right)}^{2}}\right)\right),$$

where *c* represents the *c*th GC, *c* = 1, *C*. where ${\bar{w}}_{c}$, ${\bar{\mu }}_{c,1}$, and ${\bar{\Sigma }}_{c,11}$ are the average of weight, mean vector, and covariance matrix, respectively, of the *c*th component among all of the 100 parameters sets of MdUI-GMM. *i* represents the *i*th modeling process, *i* = 1, 2, …, 100.

Table 2 shows the calculated SD of a uniform initialization process. The number of GCs is set to be *C* = 2 to cover the GW feature sample set. It is observed that the values of SD are all less than 10^−15^, which can be considered to be zero. Hence, it can be concluded that the modeling results of the MdUI-GMM are always stable. 

Table 3 shows the calculated SD of a *k*-means initialization process. The number of GCs is set to be *C* = 2 to cover the GW feature sample set. It is observed that the values of SD are higher than those of the MdUI-GMM, which means that the *k*-means initialization method is relatively less stable than the uniform initialization method.

### 3.4. Calibration between PMI and Crack Length Using Prior Specimens

**Figure 13:**
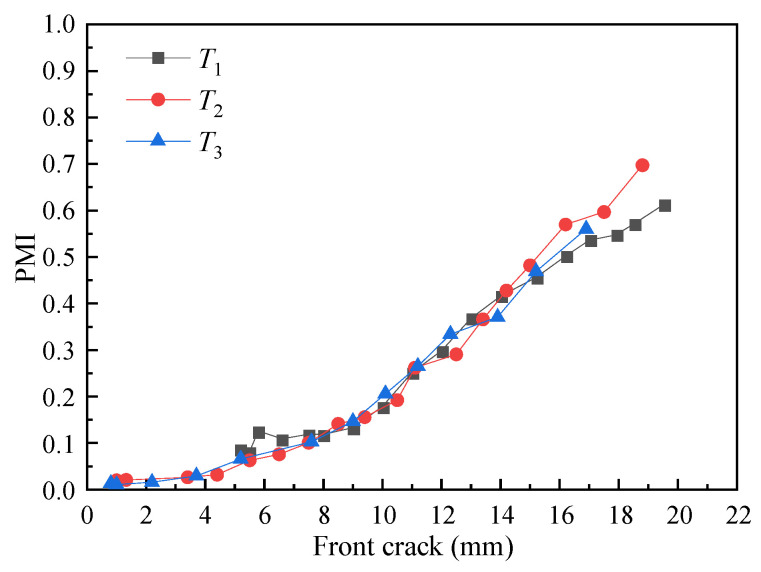
Variations of PMI with front crack length.

The first three specimen *T*_1_–*T*_3_ are used as prior samples for quantitative calibration, and *T*_4_ is used for testing. For *T*_1_–*T*_3_, the GW features of channel 2-1, channel 2-3, and channel 4-1 are fused to construct MdUI-GMM. During the monitoring process, PMI between monitoring MdUI-GMM and baseline MdUI-GMM is calculated to realize quantitative calibration of front crack length. The variation of PMIs with the length of the front crack for *T*_1_–*T*_3_ are shown in Figure 13.

For *T*_1_–*T*_3_, the relationship between PMI and crack length is smoothed by a second-order polynomial and then averaged. After that, the quantitative calibration relationship between PMI and the front crack length is established.

**Figure 14:**
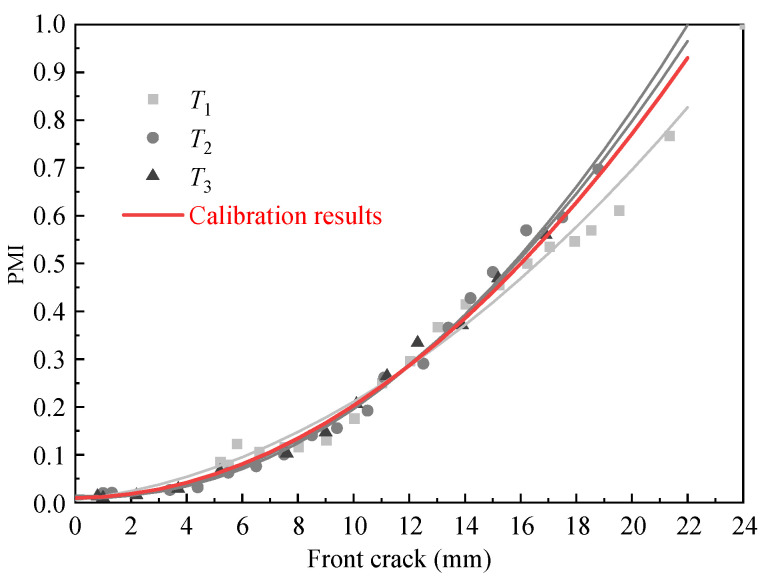
Calibration between PMI and crack length using prior specimens.

As shown in Figure 14, the equation between PMI and the front crack length is obtained as Equation (18)

(18) $$PMI=0.0019\times l^{2}+0.0006\times l+0.0095,$$

where *l* represents the front crack length.

### 3.5. Online MdUI-GMM Crack Propagation Monitoring Result

**Figure 15:**
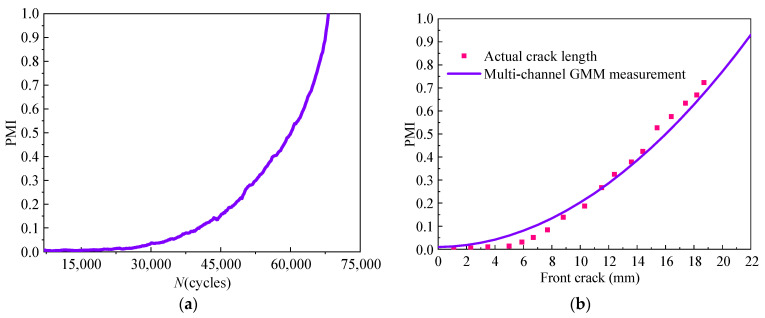
Variation of PMI during fatigue load and crack quantification results of *T*_4_. (**a**) Variation of PMI with the number of load cycles. (**b**) Results of the MdUI-GMM measurement of the crack length.

During the loading process, the GW signal is obtained online. Then, the monitoring MdUI-GMM can be established. Next, PMI between monitoring MdUI-GMM and baseline MdUI-GMM can be calculated. Through the calculated PMI, combined with the calibration results above, the front crack length of *T*_4_ can be output in real-time. For *T*_4_, the variation of PMI during fatigue loading cycles is shown in Figure 15a. Results of the MdUI-GMM measurement of the crack length are in good agreement with the actual crack length, as illustrated in Figure 15b.

The maximum absolute error of MdUI-GMM online crack length measurement is 3.4 mm and the minimum absolute error is 0.1 mm, are illustrated in Table 4.

It can be noted that on-line crack quantification can be realized by measuring PMI between monitoring MdUI-GMM and baseline MdUI-GMM. MdUI-GMM based SHM method can be used for on-line crack monitoring without aircraft grounding or structural disassembly and inspection.

### 3.6. Comparison between Single-Channel and Multi-Channel Fusion

**Figure 16:**
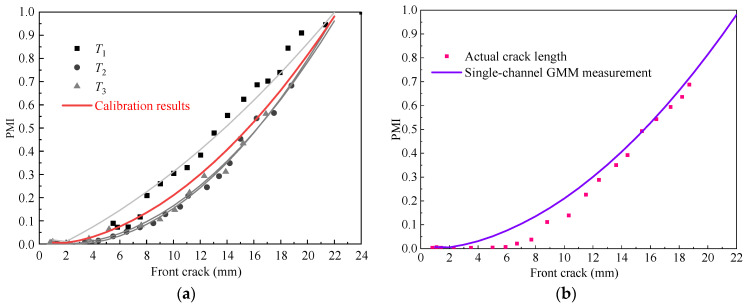
Single-channel GMM based crack quantification results of *T*_4_. (**a**) Calibration relationship between PMI and the front crack length using prior specimens. (**b**) Crack quantification results of *T*_4_.

The calibration relationship between PMI and the crack length using single-channel GMM is shown in Figure 16a. Results of the single-channel GMM based online crack quantification and actual crack length of *T*_4_ are illustrated in Figure 16b. The actual crack length, MdUI-GMM based online crack quantification results, and the absolute error of diagnosis are illustrated in Table 5.

Compared with Figure 14 and Figure 16a, the scatter of the relationship between PMI and crack length of single-channel is higher than multi-channel. In addition, as shown in Table 5, the square sum of the error of multi-channel GMM measurement is smaller than that of single-channel GMM. From this, it is clear that multi-channel fusion improves the quantification compared to single-channel measurement. This approach is quite promising for use in engineering applications.

## 4. Conclusions

In this paper, a MdUI-GMM is proposed to improve the accuracy and stability of crack quantification under uncertainty. This method integrates multi-channel GW features to establish multi-dimensional GMM considering that different GW channels have different sensitivity to crack. Moreover, a uniform initialization method is adopted to improve the constructing stability of multi-dimensional GMM. In addition, the relationship between the PMI and crack length is calibrated with fatigue tests on prior specimens. Finally, the proposed method is applied for online crack quantification on notched specimens of an aircraft spar with complex fan-shaped cracks under uncertainty. By integrating the multi-channel GW features, the square sum of the error is reduced from 38.57 to 23.30. The results show that multi-channel fusion improves the quantification compared to the single-channel measurement of the crack length. Moreover, the standard deviations of the MdUI-GMM parameter sets are all less than 10^−15^, which is much smaller than the traditional *k*-means initialization method when performing 100 times. The results show that the construction results of the uniform initialization GMM are always stable.

## Figures and Tables

**Table 1:** The mechanical properties of 7A04.

Material	Yield Strength (MPa)	Young Modulus (GPa)	Tensile Strength (MPa)
7A04	420	70	530

**Table 2:** Standard deviation (SD) of a uniform initialization process performing 100 times.

Different States of Structure		SD_w_	SD_μ_	SD_Σ_
Baseline	GC 1	9.9920 × 10^−17^	4.1633 × 10^−18^	5.0963 × 10^−21^
	GC 2	0	3.7035 × 10^−18^	6.8779 × 10^−19^
1–2 mm	GC 1	0	1.3878 × 10^−18^	9.4567 × 10^−20^
	GC 2	2.7756 × 10^−18^	2.8343 × 10^−18^	7.1866 × 10^−20^
2–4 mm	GC 1	1.2212 × 10^−16^	1.5266 × 10^−17^	8.2030 × 10^−20^
	GC 2	1.1102 × 10^−16^	3.0687 × 10^−18^	4.3368 × 10^−20^
4–6 mm	GC 1	4.4409 × 10^−17^	1.1102 × 10^−17^	5.1575 × 10^−20^
	GC 2	9.9920 × 10^−17^	4.6623 × 10^−18^	1.1132 × 10^−19^

**Table 3:** SD of a *k*-means initialization process performing 100 times.

Different States of Structure		SD_w_	SD_μ_	SD_Σ_
Baseline	GC 1	0.0235	0.0162	5.0064 × 10^−5^
	GC 2	0.0206	0.0049	1.7724 × 10^−5^
1–2 mm	GC 1	0.0090	0.0018	2.6628 × 10^−5^
	GC 2	0.0090	3.4787 × 10^−4^	2.1773 × 10^−5^
2–4 mm	GC 1	0.0224	0.0027	2.2470 × 10^−5^
	GC 2	0.0224	0.0015	2.5271 × 10^−5^
4–6 mm	GC 1	0.0249	0.0037	3.5035 × 10^−5^
	GC 2	0.0249	9.0958 × 10^−4^	2.3603 × 10^−5^

**Table 4:** Results of MdUI-GMM based crack quantification and errors of *T*_4._

PMI	Actual Crack Length (mm)	MdUI-GMM Measurement of the Crack Length (mm)	Absolute Error
0.015	5.0	1.6	3.4
0.050	6.7	4.5	2.2
0.080	7.7	6.0	1.7
0.140	8.8	8.2	0.6
0.190	10.3	9.7	0.6
0.270	11.5	11.6	0.1
0.330	12.4	12.9	0.5
0.380	13.6	13.9	0.3
0.420	14.4	14.6	0.2
0.530	15.4	16.5	1.1
0.580	16.4	17.3	0.9
0.630	17.4	18.0	0.6
0.670	18.2	18.6	0.4
0.720	18.7	19.3	0.6

**Table 5:** Results of single-channel GMM based crack quantification and errors of *T*_4._

PMI	Actual (mm)	Single-Channel GMM (mm)	Absolute Error of Single-Channel GMM	Absolute Error of Multi-Channel GMM
0.015	5.2	0.6	4.6	3.4
0.050	7.0	4.9	2.1	2.2
0.080	8.1	6.2	1.9	1.7
0.140	9.8	8.1	1.7	0.6
0.190	11.0	9.5	1.5	0.6
0.270	12.6	11.4	1.2	0.1
0.330	13.6	12.6	1.0	0.5
0.380	14.4	13.5	0.9	0.3
0.420	15.0	14.2	0.8	0.2
0.530	16.5	16.1	0.4	1.1
0.580	17.1	16.8	0.3	0.9
0.630	17.8	17.5	0.3	0.6
0.670	18.2	18.1	0.1	0.4
0.720	18.8	18.7	0.1	0.6
The square sum of the error			38.57	23.30

## Data Availability

Not applicable.
